# Which psychotherapy is effective in panic disorder? Findings and reflections from a systematic network meta-analysis

**DOI:** 10.1192/j.eurpsy.2021.336

**Published:** 2021-08-13

**Authors:** D. Papola, G. Ostuzzi, C. Gastaldon, M. Purgato, C. Del Giovane, A. Pompoli, E. Karyotaki, M. Sijbrandij, T. Furukawa, P. Cuijpers, C. Barbui

**Affiliations:** 1 Department Of Neuroscience, Biomedicine And Movement Sciences, University of Verona, Verona, Italy; 2 Neuroscience, Psychological And Psychiatric Science, Science Of Bio Movement, University of Verona, Verona, Italy; 3 Institute Of Primary Health Care (biham), University of Bern, Bern, Switzerland; 4 -, Psychiatric Rehabilitation Clinic Villa San Pietro, Trento, Italy; 5 Department Of Clinical, Neuro And Developmental Psychology, Vrije Universiteit, Amsterdam, Netherlands; 6 Departments Of Health Promotion And Human Behavior, Kyoto University, Kyoto, Japan

**Keywords:** network meta-analysis, panic disorder, psychotherapy

## Abstract

**Introduction:**

Panic disorder is among the most prevalent anxiety diseases. Although psychotherapy is recommended as first-line treatment for panic disorder, little is known about the relative efficacy of different types of psychotherapies.

**Objectives:**

To evaluate the effectiveness and acceptability of different types of psychotherapies for adults suffering from panic disorder, with or without agoraphobia.

**Methods:**

We are conducting a systematic network meta-analysis of randomized controlled trials examining panic disorder. A comprehensive search was performed to identify relevant studies. The primary efficacy outcome is anxiety symptoms at study endpoint. The primary acceptability outcome is all-cause trial discontinuation at endpoint. Pairwise and network meta-analysis will be conducted. We are considering any kind of psychotherapy delivered by any therapist, as long as they were trained to deliver the therapy, or as self-help.

**Results:**

To date we have identified 126 panic disorder and agoraphobia trials. The publication time span ranges from 1968 to 2020. We are now extracting data to provide an overview of the included study characteristics. The statistical analysis will be conducted between December 2020 and January 2021, and its results presented for the first time at the forthcoming 2021 EPA congress.

**Conclusions:**

126 trials on psychotherapy for panic disorders in adults are available. Because of this huge body of knowledge, it is important that the results of these studies are summarized using network meta-analytic techniques. The findings of this study will guide future research as knowledge gaps will be easily identified. Moreover, policymakers will have the opportunity to use this summarized knowledge to inform evidence-based decision making.

**Disclosure:**

No significant relationships.

